# One-Anastomosis Gastric Bypass (OABG) vs. Single Anastomosis Duodeno-Ileal Bypass (SADI) as revisional procedure following Sleeve Gastrectomy: results of a multicenter study

**DOI:** 10.1007/s00423-024-03306-y

**Published:** 2024-04-16

**Authors:** Pierpaolo Gallucci, Giuseppe Marincola, Francesco Pennestrì, Priscilla Francesca Procopio, Francesca Prioli, Giulia Salvi, Luigi Ciccoritti, Francesco Greco, Nunzio Velotti, Vincenzo Schiavone, Antonio Franzese, Federica Mansi, Matteo Uccelli, Giovanni Cesana, Mario Musella, Stefano Olmi, Marco Raffaelli

**Affiliations:** 1 Centro Dipartimentale Di Chirurgia Endocrina E Dell’Obesità, U.O.C. Chirurgia Endocrina E Metabolica, Fondazione Policlinico Universitario Agostino Gemelli IRCCS, Rome, Italy; 2 Centro Di Ricerca In Chirurgia Delle Ghiandole Endocrine E Dell’Obesità, Università Cattolica del Sacro Cuore, Rome, Italy; 3 Dipartimento Di Scienze Biomediche Avanzate, Ospedale Universitario Federico II, Università Degli Studi Di Napoli Federico II, Naples, Italy; 4 Dipartimento Di Chirurgia Generale Ed Oncologica, Centro Di Chirurgia Laparoscopica E Bariatrica, Policlinico San Marco, Gruppo San Donato, Zingonia, BG Italy; 5 University Vita-Salute San Raffaele, Milan, Italy

**Keywords:** Revisional surgery, One-Anastomosis Gastric Bypass (OAGB), Single Anastomosis Duodeno-Ileal Bypass (SADI), Complications, Suboptimal clinical response, Recurrence of weight

## Abstract

**Purpose:**

Sleeve Gastrectomy (SG) is the most performed bariatric surgery, but a considerable number of patients may require revisional procedures for suboptimal clinical response/recurrence of weight (SCR/RoW). Conversion options include One-Anastomosis Gastric Bypass (OAGB) and Single Anastomosis Duodeno-Ileal Bypass (SADI). The study aims to compare SADI vs. OAGB as revisional procedures in terms of early and mid-term complications, operative time, postoperative hospital stay and clinical outcomes.

**Methods:**

All patients who underwent OAGB or SADI as revisional procedures following SG for SCR/RoW at three high-volume bariatric centers between January 2014 and April 2021 were included. Propensity score matching (PSM) analysis was performed. Demographic, operative, and postoperative outcomes of the two groups were compared.

**Results:**

One hundred and sixty-eight patients were identified. After PSM, the two groups included 42 OAGB and 42 SADI patients. Early (≤ 30 days) postoperative complications rate did not differ significantly between OAGB and SADI groups (3 bleedings vs. 0, *p* = 0.241). Mid-term (within 2 years) complications rate was significantly higher in the OAGB group (21.4% vs. 2.4%, *p* = 0.007), mainly anastomotic complications and reflux disease (12% of OAGBs). Seven OAGB patients required conversion to another procedure (Roux-en-Y Gastric Bypass—RYGB) vs. none among the SADI patients (*p* = 0.006).

**Conclusions:**

SADI and OAGB are both effective as revisional procedures for SCR/RoW after SG. OAGB is associated with a significantly higher rate of mid-term complications and a not negligible rate of conversion (RYGB). Larger studies are necessary to draw definitive conclusions.

## Introduction

Sleeve Gastrectomy (SG) has gained popularity as a safe and effective bariatric procedure, leading to significant short-term weight loss and improvements in obesity-related comorbidities [1, 2]. Originally developed in the 1990s as part of the Biliopancreatic Diversion with Duodenal Switch (BPD/DS) procedure, SG was afterwards introduced as primary bariatric surgery, rapidly gaining popularity. To date, SG represents the most commonly performed bariatric operation worldwide [2, 3].

However, this bariatric procedure is not without limitations. In the long term, 15 to 50% of patients may experience suboptimal clinical response (SCR) or recurrence of weight (RoW) [4–7]. Additionally, refractory gastroesophageal reflux disease (GERD) may potentially prompt conversion procedure [8, 9].

The potential options for revisional surgery following SG include: Roux-en-Y Gastric Bypass (RYGB), One-Anastomosis Gastric Bypass (OAGB), BPD/DS, Single Anastomosis Duodeno-Ileal Bypass (SADI) [10–12].

BPD/DS offers excellent long-term weight loss outcomes. However, this complex procedure is associated with a higher risk of long-term nutritional deficiencies [13, 14].

OAGB has been identified as a reliable and feasible revisional procedure after SG. It offers promising outcomes, including a simple technique, good long-term weight loss and improved metabolic parameters, but bile reflux and anastomotic ulcer represent potential procedure-related issues, ranging from 7.8 to 55.5% [15–17] and 0.47 to 7.35%, respectively [18].

SADI is a simpler surgical procedure compared to BPD/DS. It requires one anastomosis, which may result in a shorter operative time and a potentially reduced risk of surgical complications, while maintaining similar outcomes in terms of weight loss and comorbidities resolution [19].

While several studies investigated the outcomes of OAGB and SADI as revisional procedures following SG, further comparative analyses are necessary to guide clinical decision-making. The present multi-institutional retrospective study aims to evaluate and compare the peri-operative and post-operative outcomes, as well as the short-term and long-term complications associated with OAGB and SADI as conversion procedures following SG.

## Materials and methods

A multi-center observational study was conducted from January 2014 to April 2021 at three Italian Referral Centers for Bariatric Surgery: (A) U.O.C. Chirurgia Endocrina e Metabolica, Fondazione Policlinico Universitario Agostino Gemelli IRCCS, Rome; (B) Dipartimento di Scienze Biomediche Avanzate, Federico II University, Naples; (C) Dipartimento di Chirurgia Generale ed Oncologica, Policlinico San Marco, Zingonia.

Data from all patients scheduled for bariatric surgery were prospectively collected in a specifically designed and de-identified database (Microsoft Excel, Microsoft Corporation, Redmond, WA, USA).

Study population: From January 2014 to April 2021, a total of 17,339 patients underwent bariatric surgical procedures.

Patients were considered eligible for the present study if scheduled for OAGB or SADI as a revisional surgery after SG and completed 2 years of follow-up after second operation.

Exclusion criteria included open bariatric procedures, primary surgery different from SG, revisional surgery different from OAGB and SADI.

All patients underwent esophagogastroduodenoscopy prior to revision surgery. In case of suspicious GERD, 24-h pH-metry was performed.

Criteria for revisional bariatric surgery included RoW and SCR. Specifically, RoW was defined by a post-operative weight increase ≥ 50% of the lowest weight achieved after the initial surgery; SCR was defined as %EWL < 50% at the post-operative nadir weight.

Demographic, clinical, and outcome characteristics of the included patients were registered and compared between the groups. The list of registered parameters is reported in Table 1.

**Table 1:** Comparative analysis of the clinicopathological characteristics of all patients who met the inclusion criteria: OAGB (*n* = 126) vs. SADI (*n* = 42)

Variable	OAGB *N* = 126	SADI *N* = 42	*p* value
Age, years (mean ± SD)	46.1 ± 9.4	42.9 ± 9.7	0.066
Gender, (M:F)	21:105	9:33	0.485
BMI, kg/m^2^ (mean ± SD)	38.9 ± 7.4	44.8 ± 8.3	0.001
Weight, kg (mean ± SD)	100.2 ± 15.6	124.2 ± 8.4	0.001
Comorbidity, (*n*, %)	49 (38.9%)	19 (45.2%)	0.468
OSAS, (*n*, %)	14 (11.1%)	10 (23.8%)	0.042
Hypertension, (*n*, %)	40 (31.7%)	18 (42.8%)	0.191
Type 2 diabetes mellitus, (*n*, %)	11 (8.7%)	8 (18.9%)	0.067
Previous abdominal surgery			0.001
Laparoscopic, (*n*, %)	116 (91.9%)	24 (57.1%)	
Open, (n, %)	10 (7.9%)	18 (42.8%)	
Age at SG, years (mean ± SD)	41.6 ± 9.9	38.4 ± 9.8	0.081
BMI at SG, kg/m^2^ (mean ± SD)	43.9 ± 4.6	50.7 ± 8.4	0.001
Weight at SG, kg (mean ± SD)	113.1 ± 12.5	140.5 ± 8.7	0.001
%TWL after SG (mean ± SD)	14.4 ± 6.0	10.1 ± 9.5	0.001
Time to previous SG, months (mean ± SD)	54.5 ± 32.1	68.5 ± 43.6	0.027
Indication to revisional procedure			0.001
SCR, (*n*, %)	24 (19.1%)	32 (76.2%)	
RoW, (*n*, %)	102 (80.9%)	10 (23.8%)	

*OAGB* One-Anastomosis Gastric Bypass; *SADI* Single Anastomosis Duodeno–Ileal Bypass; *SD* standard deviation; *IQR* 75% interquartile range; *BMI* body mass index; *OSAS* obstructive sleep apnea syndrome; *SG* Sleeve Gastrectomy; *SCR* suboptimal clinical response; *RoW* recurrence of weight; *%TWL* total wight loss

The follow-up was conducted through outpatient consultations with a multidisciplinary team at 30 days, 3 months, 6 months, 12 months, and 24 months after surgery. The follow-up evaluation concluded on the 1st May 2023.

The patients included in our study adhered to the guidelines provided by the Italian Society of Bariatric Surgery and Metabolic Disorders (SICOb) [20].

The selection of the surgical approach for each patient was primarily based on the operating surgeon’s preference, in line with national guidelines.

The details of the surgical procedures have been previously reported [21–23]. Delving deeper in this topic, for SADI procedure, the length of common channel was approximately 300 cm from the ileocecal valve. On the other hand, in the OAGB the length of the biliopancreatic limb varied from 150 to 250 cm from the Treitz’s ligament, considering the surgeon’s preferences and the weight loss goals to be achieved [24–26].

Weight loss efficacy was assessed through %EWL and %TWL, both in percentages and in kilograms, using standard formula: %EWL = [(baseline weight − postoperative weight)/(baseline weight − ideal body weight)] × 100. Ideal body weight was calculated using the weight equivalent to a BMI of 25 kg/m^2^; %TWL = (baseline weight/baseline weight − postoperative weight) × 100.

This study was conducted following approval by the local ethical committee, in accordance with the ethical standards of the institutional research committee and with the 1964 Helsinki declaration and its later amendments or comparable ethical standards.

### End points

The primary endpoint of the study was to compare early and mid-term (between 30 days and 2 years) complications rate between SADI and OAGB. The secondary endpoint consisted in the evaluation of conversions rate to RYBG and the comparison of the two approaches in terms of clinical outcome, with special regard to %EWL and %TWL.

### Statistical analysis

Statistical analysis was performed by means of propensity score matching (PSM) using the “1:1 nearest neighbor” matching method with a caliper of 0.01, where the type of surgical procedure (OAGB or SADI) was used as the binary independent variable in the regression model of the propensity score. Key variables for PSM were gender, BMI at revisional surgery, age, type of surgery, and associated comorbidities (type 2 diabetes mellitus—T2DM, hypertension, obstructive sleep apnea syndrome—OSAS).

To compare foundational traits and pre- and post-surgery factors, a bivariate examination was used. The Shapiro–Wilks test gauged normal distribution. For categorical factors comparison, Fisher’s exact test and the Chi-square test were applied. Continuous data were denoted as either median (IQR) or mean (± SD) and based on the data distribution in the study group, either a paired sample *t* test or Mann–Whitney *U* test was used for comparison.

Essential demographic and medical details were sourced from patient records and digital databases. Statistical analysis and PSM were conducted using Stata version 17.0 (StataCorp, College Station, TX, USA). All analyses were two-tailed and the threshold for statistical significance was set at *p* < 0.05.

## Results

### Study population

Between January 2014 and April 2021, 2446 revisional bariatric surgeries were performed at the three participating centers. Among them, 168 patients met the specific inclusion criteria for our study. From this subset, 126 underwent OAGB procedure, and 42 underwent SADI procedure. After PSM, the study groups consisted of 84 patients, with 42 patients in each group.

### Demographics and postoperative outcomes

Table 1 shows a comparative analysis of the demographic features and comorbidities of all patients who met the inclusion criteria.

No significant differences were observed between the SADI and OAGB groups in terms of age, gender distribution, hypertension, and T2DM (*p* > 0.05).

The mean BMI before revisional surgery was 38.9 ± 7.4 kg/m^2^ and 44.8 ± 8.2 kg/m^2^ for the OAGB and the SADI groups, respectively (*p* = 0.001). The mean BMI at the time of SG was 43.9 ± 4.6 kg/m^2^ in the OAGB group and 50.7 ± 8.4 kg/m^2^ in the SADI group (*p* = 0.001). Moreover, the time frame since the previous SG was longer in the SADI group compared to the OAGB group (68.5 ± 43.6 vs. 54.5 ± 32.1 months; *p* = 0.027). OSAS prevalence was also higher in the SADI group (*p* = 0.042). Additionally, the SADI group had a more pronounced prevalence of SCR (76.2% vs. 19.1%) compared to the OAGB group (*p* = 0.001).

Table 2 presents a comparison of the intra-operative and post-operative data between the two groups. The OAGB group had a longer median post-operative hospital stay (4 days vs. 2 days, *p* = 0.001). The SADI group had no 30-day post-operative complications, differently from the OAGB group patients, experiencing bleeding (9 patients, 7.1%), pneumonia (1 patient, 0.8%), and deep vein thrombosis (2 patients, 1.6%). Therefore, the analysis of the unmatched group showed a significant difference concerning the early postoperative complications rate (12.7% for OAGB vs. 0% for SADI; *p* = 0.015). Among early complications, emergency surgical re-operation was necessary for 4 (3.2%) patients in the OAGB group, while no re-operation was observed in the SADI group (*p* = 0.243). Mid-term complications were significantly higher in the OAGB group (31.7% vs. 2.4%, *p* = 0.007). Delving deeper, specific complications reported in the OAGB group included internal hernia (4 patients, 3.2%), twisting of the small bowel (4 patients, 3.2%), anastomotic ulcer (9 patients, 7.2%), anastomotic stenosis (8 patients, 6.3%), malnutrition (1 patient, 0.8%), and GERD (7 patients, 5.5%). At 2-year follow-up, 6 (4.8%) OAGB patients developed RoW. In contrast, none of these complications were observed in the SADI group, except only one (2.4%) case of twisting of the small bowel.

**Table 2:** Comparative analysis of intraoperative and postoperative data of all patients who met the inclusion criteria: OAGB (n = 126) vs SADI (n = 42)

Variable	OAGB *N* = 126	SADI *N* = 42	p-value
Approach			0.014
Laparoscopic (*n*, %)	126 (100%)	40 (95.2%)	
Robotic (*n*, %)	0	2 (4.8%)	
Post-operative ICU (*n*, %)	4 (3.2%)	0	0.573
Post-operative hospital stay, days (median, IQR)	4 (3 – 4)	2 (2 – 3)	0.001
30th day post-operative complications (*n*, %)	16 (12.7%)	0	0.015
Reoperation (*n*, %)	4 (3.2%)	0	0.243
Pneumonia (*n*, %)	1 (0.8%)	0	0.563
Bleeding (*n*, %)	9 (7.1%)	0	0.045
Deep vein thrombosis (*n*, %)	2 (1.6%)	0	0.411
Gastric stump leakage (*n*, %)	1 (0.8%)	0	0.563
Anastomotic leakage (*n*, %)	3 (2.4%)	0	0.313
Mid-term complications (*n*, %)	40 (31.7%)	1 (2.4%)	0.001
Internal hernia (*n*, %)	4 (3.2%)	0	0.243
Twisting of small bowel (*n*, %)	4 (3.2%)	1 (2.4%)	0.793
Anastomotic ulcer (*n*, %)	9 (7.2%)	0	0.045
Anastomotic stenosis (*n*, %)	8 (6.3%)	0	0.075
Malnutrition (*n*, %)	1 (0.8%)	0	0.563
RoW at 2 years follow-up (*n*, %)	6 (4.8%)	0	0.338
GERD (*n*, %)	7 (5.5%)	0	0.194
Converted to RYGB (*n*, %)	32 (25.4%)	0	0.001

*OAGB* One-Anastomosis Gastric Bypass; *SADI* Single Anastomosis Duodeno–Ileal Bypass; *SD* standard deviation; *IQR* 75% interquartile range; *SG* Sleeve Gastrectomy; *RoW* recurrence of weight; *GERD* gastroesophageal reflux disease; *ICU* intensive care unit; *RYGB* Roux-en-Y Gastric Bypass

At center A, OAGB biliary limb length has been standardized to 200 cm from the Treitz’s ligament. Differently, the mean biliary limb length has been 190.2 ± 10.5 cm at center B and 220 ± 8.5 cm at center C.

### Post-PSM results

Table 3 presents demographic and clinical features after PSM analysis. There were no significant differences concerning age, gender distribution, and comorbidities (*p* > 0.05). The SADI group had a notably higher BMI at the time of SG (*p* = 0.001) and a higher incidence of SCR (71.4% vs. 16.7%) compared to the OAGB group. However, the OAGB group exhibited a significantly higher prevalence of RoW (83.3% vs. 28.6%) post-SG (*p* = 0.001). The mean length of the biliopancreatic limb for OAGB was 231.3 ± 48.3 cm. During SADI procedures, a re-sleeve was never performed. A notable difference was observed in the mean pre-operative weight of patients before revisional surgery (118.5 ± 9.2 kg in the OAGB group vs. 124.2 ± 8.4 kg for the SADI group; *p* = 0.002).

**Table 3:** Demographic characteristics between OAGB group (*n* = 42) and SADI group (*n* = 42) after propensity matching score analysis

Variable	OAGB *N* = 42	SADI *N* = 42	*p* value
Age, years (mean ± SD)	41.2 ± 9.8	42.9 ± 9.7	0.378
Gender, (M:F)	15:27	9:33	0.146
BMI, kg/m^2^ (mean ± SD)	43.8 ± 6.6	44.8 ± 8.2	0.543
Weight, kg (mean ± SD)	118.5 ± 9.2	124.2 ± 8.4	0.002
Comorbidity (*n*, %)	21 (50%)	19 (45.2%)	0.468
OSAS (*n*, %)	7 (16.7%)	10 (23.8%)	0.418
Hypertension (*n*, %)	20 (47.6%)	18 (42.8%)	0.663
Type 2 diabetes mellitus (*n*, %)	5 (11.9%)	8 (18.9%)	0.364
Previous abdominal surgery			0.064
Laparoscopic (*n*, %)	32 (76.2%)	24 (57.1%)	
Open (*n*, %)	10 (23.8%)	18 (42.8%)	
Age at SG, years (mean ± SD)	35.9 ± 10.6	38.4 ± 9.8	0.298
BMI at SG, kg/m^2^ (mean ± SD)	43.9 ± 4.6	50.7 ± 8.4	0.001
Weight at SG, kg (mean ± SD)	118.8 ± 10.1	140.5 ± 8.7	0.001
Time to previous SG, months (mean ± SD)	61.8 ± 31.2	68.5 ± 43.6	0.426
Indication to revisional procedure			0.001
SCR (*n*, %)	7 (16.7%)	30 (71.4%)	
RoW (*n*, %)	35 (83.3%)	12 (28.6%)	

*OAGB* One-Anastomosis Gastric Bypass; *SADI* Single Anastomosis Duodeno–Ileal Bypass; *SD* standard deviation; *IQR* 75% interquartile range; *BMI* body mass index; *OSAS* obstructive sleep apnea syndrome; *SG* Sleeve Gastrectomy; *SCR* suboptimal clinical response; *RoW* recurrence of weight; *%TWL* total wight loss

Table 4 compares intra-operative and post-operative data between groups after PSM. The SADI group experienced a shorter postoperative hospital stay with a median of 2 days (IQR 2–3) compared to the OAGB group’s median of 4 days (IQR 4–5) (*p* = 0.001). The OAGB group showed a 7.1% of early complication rate due to post-operative bleeding, while for none of the SADI group patients complications were reported, though with a non-significant difference (*p* = 0.241).

**Table 4:** Intraoperative and postoperative outcomes between OAGB group (*n* = 42) and SADI group (*n* = 42) after propensity matching score analysis

Variable	OAGB *N* = 42	SADI *N* = 42	*p* value
Approach			0.152
Laparoscopic (*n*, %)	42 (100%)	40 (95.2%)	
Robotic (*n*, %)	0	2 (4.8%)	
Post-operative ICU (*n*, %)	4 (9.5%)	0	0.041
Post-operative hospital stay, days (median, IQR)	4 (4–5)	2 (2–3)	0.001
30th day post-operative complications (*n*, %)	3 (7.1%)	0	0.241
Reoperation (*n*, %)	0	0	1
Pneumonia (*n*, %)	0	0	1
Bleeding (*n*, %)	3 (7.1%)	0	0.241
Deep vein thrombosis (*n*, %)	0	0	1
Gastric stump leakage (*n*, %)	0	0	1
Anastomotic leakage (*n*, %)	0	0	1
Mid-term complications (*n*, %)	9 (21.4%)	1 (2.4%)	0.007
Internal hernia (*n*, %)	1 (2.4%)	0	0.314
Twisting of small bowel (*n*, %)	1 (2.4%)	1 (2.4%)	1
Anastomotic ulcer (*n*, %)	2 (4.8%)	0	0.262
Anastomotic stenosis (*n*, %)	2 (4.8%)	0	0.262
Malnutrition (*n*, %)	1 (2.4%)	0	0.314
RoW at 2 years follow-up (*n*, %)	1 (2.4%)	0	0.314
GERD (*n*, %)	1 (2.4%)	0	0.314
Converted to RYGB (*n*, %)	7 (16.7%)	0	0.006

*OAGB* One-Anastomosis Gastric Bypass; *SADI* Single Anastomosis Duodeno–Ileal Bypass; *SD* standard deviation; *IQR* 75% interquartile range; *SG* Sleeve Gastrectomy; *RoW* recurrence of weight; *GERD* gastroesophageal reflux disease; *ICU* intensive care unit; *RYGB* Roux-en-Y Gastric Bypass

Moreover, mid-term complications rates were 21.4% and 2.4% in OAGB group and SADI group, respectively (*p* = 0.007).

Within the OAGB cohort, specific complications like internal hernia, twisting of the small bowel, malnutrition and GERD occurred in 1 (2.4%) case each. In OAGB group, 2 (4.8%) anastomotic ulcers and 2 (4.8%) anastomotic stenosis were observed. Additionally, the OAGB group presented a higher conversion rate to RYGB (16.7% vs. 0% of the SADI group, *p* = 0.006) following revisional surgery.

### Weight loss outcomes

Patients who underwent OAGB showed a significantly lower %EWL at follow-up evaluation compared to SADI group, both in the complete series (53.3 ± 18.7% vs. 72.3 ± 26.9% at 1-year follow-up and 48.9 ± 18.7% vs. 90.2 ± 24.4% at 2-year follow-up) and also after PSM (48.6 ± 26.9% vs. 72.3 ± 26.9% at 1-year follow-up and 62.5 ± 17.7% vs. 90.2 ± 24.4% at 2-year follow-up). After PSM, at 1-year follow-up, the mean %TWL for the SADI group was 31.2 ± 11.7%, compared to 26.5 ± 10.9% for the OAGB group, though without significant difference (*p* = 0.059). At 2-year follow-up, the SADI group’s mean %TWL increased to 41.7 ± 10.2%, which was significantly greater than the OAGB group’s mean 31.6 ± 10.7% (*p* = 0.001). Bariatric outcomes are showed in Tables 5 and 6.

**Table 5:** Follow-up data after 1 and 2 years of all patients who met the inclusion criteria: OAGB (*n* = 126) vs. SADI (*n* = 42)

Variable	OAGB *N* = 126	SADI *N* = 42		*p* value	
1 year follow-up					
BMI, kg/m^2^ (mean ± SD)	30.2 ± 4.9		30.9 ± 6.4		0.512
%EWL, (mean ± SD)	53.3 ± 18.7		72.3 ± 26.9		0.018
%TWL, (mean ± SD)	22.4. ± 9.2		31.2 ± 11.7		0.001
2 years follow-up					
BMI, kg/m^2^ (mean ± SD)	29.1 ± 4.5		26.1 ± 6.2		0.135
%EWL, (mean ± SD)	48.9 ± 18.7		90.2 ± 24.4		0.003
%TWL, (mean ± SD)	25.2 ± 8.6		41.7 ± 10.2		0.001

*OAGB* One-Anastomosis Gastric Bypass; *SADI* Single Anastomosis Duodeno–Ileal bypass; *SD* standard deviation; *BMI* body mass index; *%EWL* percentage of excess weight loss; *%TWL* total wight loss

**Table 6:** Follow-up data: OAGB (*n* = 42) vs. SADI (*n* = 42) after PSM

Variable	OAGB *N* = 42	SADI *N* = 42		*p* value	
1 year follow-up					
BMI, kg/m^2^ (mean ± SD)	32.2 ± 3.5		30.9 ± 6.4		0.475
%EWL, (mean ± SD)	48.6 ± 26.9		72.3 ± 26.9		0.001
%TWL, (mean ± SD)	26.5 ± 10.9		31.2 ± 11.7		0.059
2 years follow-up					
BMI, kg/m^2^ (mean ± SD)	29.9 ± 2.2		26.1 ± 6.2		0.001
%EWL, (mean ± SD)	62.5 ± 17.7		90.2 ± 24.4		0.001
%TWL, (mean ± SD)	31.6 ± 10.7		41.7 ± 10.2		0.001

*OAGB* One-Anastomosis gastric Bypass; *SADI* Single Anastomosis Duodeno–Ileal Bypass; *SD* standard deviation; *BMI* body mass index; *%EWL* percentage of excess weight loss; *%TWL* total weight loss

## Discussion

This retrospective multi-institutional cohort study compares OAGB and SADI as revisional procedures following SG for unsuccessful bariatric outcomes, such as SCR and RoW. Our results showed that the two operations are comparable in terms of early post-operative complications, though with a higher rate of mid-term complications in the OAGB group.

The appropriate management of bariatric surgery after RoW/SCR represents a challenge.

The choice between OAGB and SADI following SG is still a matter of debate. Indeed, while both procedures represent safe and feasible options to achieve weight loss, OAGB is associated with higher post-operative complications, as though it is currently considered as a less technically demanding procedure [27, 28].

In our study, the most frequently observed mid-term complication in the OAGB group was anastomotic ulcers, occurring in 7.2% of patients. This complication was absent in the SADI group.

Delving deeper, bile reflux is a noteworthy concern after OAGB, given the absence of the pyloric valve’s controlling mechanism in this altered anatomy [29–33]. However, the prevalence and significance of this phenomenon are still subjects of concern, as findings vary based on surgical techniques employed [33, 34]. Even though we did not thoroughly assess bile reflux using esophagogastroduodenoscopy and 24-h pH-metry, at least theoretically, this complication could be related to the effect of the aforementioned OAGB design [31, 35–37]. Additionally, retained gastric antrum syndrome might also contribute to the high incidence of anastomotic ulcers [38].

On the other hand, SADI, though more technically demanding, allows the regulation of the gastric contents flow into the duodenum and the acid–base homeostasis, thanks to the pylorus and the Brunner’s glands preservation [19, 39–41].

Esparham et al. [42] evaluated OAGB-associated complications in a meta-analysis including 27,775 patients from 87 studies, focusing on the need for revision surgeries due to severe reflux. Revisional surgeries exhibited a 10% incidence of new-onset GERD compared to 4% of primary surgeries. Meanwhile esophagitis was observed in 15% of patients undergoing primary OAGB, with revisional surgeries reporting a marginally higher rate of 19%, indicating an increased risk of esophageal inflammation following revisional procedures. Both primary and revisional OAGBs were related a low incidence of Barrett’s esophagus (1%), while marginal ulcers occurred in 2% of patients. Among the revisional surgery group, 6% of patients required conversion to RYGB, emphasizing the clinical impact of reflux after initial bariatric procedures. De la Cruz et al. [43] also reported that symptomatic bile reflux was significantly more common after OAGB, with 26.7% of affected patients, as opposed to only 4.8% after SADI. None of the SADI group patients developed anastomotic ulcers, differently from 2.4% of OAGB group patients. Further post-operative complications, such as obstipation, anemia, and dumping syndrome were also notably more frequent in the OAGB group compared to the SADI group (14.3% and 11.9% vs. 2.4% and 4.8%, respectively). Moreover, OAGB patients exhibited a significantly higher rate of readmission (42.9%), almost doubling that one of the SADI group (23.8%).

In our experience, at 2-year-follow-up a not negligible conversion rate in the OAGB group was reported, differently to only one conversion in the SADI group. In detail, in OAGB group we registered bile reflux-related conversion in one (2.4%) patient out of 42, while other causes of conversion included anastomotic ulcer (4.8%), stenosis (4.8%), malnutrition (2.4%), and RoW (2.4%).

Similarly, Salama et al. [44] revealed a significantly higher prevalence of complications and reoperations in the OAGB group compared to the SADI group, with superior outcomes as a revisional option for RoW after SG in the latter group. Conditions like potential GERD exacerbation (6.1%), anastomotic ulcer (6.1%), bile reflux (6.1%), and RoW (4%) were reported as main reasons for conversion to a different procedure in 5 patients in the OAGB group. In contrast, only one SADI group patient required conversion to a different procedure due to intractable GERD. On the other hand, differently from our experience, a meta-analysis of Franken et al. [45] comparing eight studies with 484 OAGBs and three studies with 150 SADIs showed a OAGB relatively safe profile, with a major complication rate of 4.5%. Major complications in SADI were observed in 9 (6%) cases, including small bowel perforation, restaple-line leak, abdominal abscess, anastomosis leak, incisional hernia, internal hernia and cholecystolithiasis.

With regard with weight loss as secondary outcome of our study, our analysis assessed the long-term efficacy of SADI versus OAGB, with a primary focus on BMI and weight loss over 1- and 2-year follow-up periods. After PSM, at 1-year follow-up, the SADI cohort exhibited a notable decline in excess weight, yielding a %EWL of 72.3 ± 26.9, in contrast to the OAGB group’s %EWL of 48.6 ± 26.9. Such difference underscores the heightened efficacy of SADI in weight reduction compared to OAGB. At 2-year follow-up, the %EWL divergence further intensified. The SADI cohort sustained its superior performance with a %EWL of 90.2 ± 24.4, markedly outpacing the OAGB group’s %EWL of 62.5 ± 17.7. This observation aligns with findings from the broader literature, with a reported notable disparity between the SADI and OAGB groups in several metrics in terms of %TWL (30.0 ± 18.4 vs. 19.4 ± 16.3, respectively) [44]. Similarly, the %EWL for the SADI group was markedly superior (66.2 ± 21.7), as opposed to the OAGB cohort’s 50.9 ± 30.6. This trend persisted in BMI reductions, with the SADI group showing a more pronounced decline of 12.2 ± 8.9, while the OAGB group presented a drop of 7.4 ± 5.7. On the other hand, a meta-analysis of 1057 patients from 20 studies who underwent revisional OAGB after SG reported a pooled mean %EWL of 65.2%, demonstrating a significant and sustained weight loss outcome [46]. However, the reported results are burdened by a high heterogeneity (*I*^2^) in the selected studies.

It is to underline that in the present study SADI was performed in patients with a higher weight compared to those undergoing OAGB. The reasons for this preference rely on the procedure’s anatomical and physiological impacts, the durability of the results, the surgeon’s preferences, and patients’ characteristics. The greater weight loss observed in the SADI group may underscore its efficiency in significant weight loss in these patients’ cohort.

The design of the present study should theoretically limit selection biases related to patients’ eligibility and surgeons’ preferences, making the comparison between the two procedures more valid. Despite our efforts to conduct blinded matching using PSM, we remain aware about the inherent limitation due to the retrospective nature of the study and the potential for unmeasured confounders that cannot be accounted for in the matching process. However, we should underline as a major limitation that only one of the recruiting centers has integrated both OAGB and SADI, differently from the other two only integrating OAGB in their clinical practice. This introduces potential biases in the choice of procedure-based and clinical protocols on the center’s preferences and expertise.

A recognized limitation of our retrospective study is that we did not measure the biliopancreatic limb during the SADI procedure or the common limb during the OAGB procedure. Clearly, a more standardized limbs calibration may play a significant role either for weight loss and for post-operative complications.

Moreover, it is to highlight significant disparities in terms of initial BMI among primary surgical procedures, and likewise, differences in comorbidities among groups that are not matched. Recognizing that SADI procedures are typically reserved for BMI > 50 patients is crucial. As a rule, patients eligible for SADI tend to present with an elevated BMI, which is closely related to a greater number of comorbidities*.* Furthermore, while the primary outcome of the study was to compare the complications rate between the two surgeries, real-world implications of these complications (like their severity and long-term consequences) might be more relevant than the sheer rate. Randomized studies with large sample size are necessary to impartially assess the outcomes of OAGB and SADI procedures, ensuring more reliable results. In order to properly consider late-occurring effects, monitoring patients for more than 2 years is paramount.

Nonetheless, the present paper has the merit to represent the first PSM-designed analysis which consents to partially overcome the limitations of the not-randomized nature of our study.

## Conclusions

Both SADI and OAGB have demonstrated efficacy as revisional interventions for SCR/RoW post-SG. However, patients undergoing OAGB exhibited a more pronounced incidence of mid-term complications and a consequential, non-trivial necessity for conversion to RYGB. Although the potential of both procedures is evident, the differences in their safety profiles cannot be overlooked.

Basing on the presented results, SADI should be proposed as primary option for treatment of RoW/SCR following SG.

Increasing the sample size and the length of follow-up is crucial to ensure more reliable results and monitor late-occurring effects. Besides clinical aspects, patients’ feedback on bile reflux and quality of life assessment is essential for a global understanding.

More extensive, randomized trials are paramount to solidify our understanding and offer conclusive insights into the optimal choice between these procedures for addressing SCR/RoW after SG.

## Data Availability

The raw data supporting the conclusions of this article will be made available by the corresponding author, without undue reservation.
